# Phylogenetic analysis of the complete mitochondrial genome of the Blomfild’s Beauty butterfly *Smyrna blomfildia* (Fabricius 1781) (Insecta: Lepidoptera: Nymphalidae: Nymphalini)

**DOI:** 10.1080/23802359.2021.1989337

**Published:** 2021-10-14

**Authors:** Mackenzie R. Alexiuk, Melanie M. L. Lalonde, Jeffrey M. Marcus

**Affiliations:** Department of Biological Sciences, University of Manitoba, Winnipeg, Canada

**Keywords:** Illumina sequencing, mitogenomics, Lepidoptera, Nymphalidae, Nymphalini

## Abstract

The Blomfild’s Beauty butterfly *Smyrna blomfildia* (Fabricius 1781) (Lepidoptera: Nymphalidae: Nymphalini) is a sexually dimorphic species found in Mexico, Central, and South America. Males are territorial and are more vibrantly colored than females. Genome skimming by Illumina sequencing allowed the assembly of a complete circular mitochondrial genome (mitogenome) of 15,149 bp from *S. blomfildia* consisting of 83.9% AT nucleotides, 13 protein-coding genes, 22 tRNAs, two rRNAs, and a control region in the typical butterfly gene order. The *S. blomfilda COX1* gene features an atypical start codon (CGA) while *ATP6, COX1, COX2, CYTB, ND1, ND3*, *ND4*, and *ND5* display partial stop codons completed by the addition of 3’ A residues to the mRNA. Bayesian phylogenetic reconstruction places *Smyrna* as a member of the tribe Nymphalini and sister to a clade containing genera *Araschnia*, *Vanessa*, *Polygonia*, and *Aglais*, which differs from its classic taxonomic placement in tribe Coeini.

The Blomfild’s Beauty butterfly, *Smyrna blomfildia* (Fabricius 1781) (Lepidoptera: Nymphalidae: Coeini), is found in Mexico, Central, and South America (Machado and Freitas 2001; Pfeiler et al. 2020). Adults display sexual dimorphism with territorial males being more vibrantly colored than females (Muyshondt and Muyshondt 1978). The color patterns forming within each wing sector (wing regions bounded by wing veins, also called wing ‘cells’ by entomologists) of *S. blomfildia* are homologous to those found in neighboring sectors, which develop independently and generally are weakly correlated with one another (Nijhout 1985). The few correlated wing sectors observed by Nijhout (1985) in *S. blomfildia* anticipated patterns of correlation later observed on the wings of other butterfly species (Monteiro et al. 2003, 2007; Kodandaramaiah 2009) that led to important discoveries regarding the developmental architecture underlying all insect wings (Abbasi and Marcus 2017; Banerjee and Monteiro 2020; McKenna et al. 2020).

The *S. blomfildia* caterpillars feed on nettle plant leaves of the genera *Urera*, *Urticastrum*, and other members of the family Urticaceae (Schaus 1884; Muyshondt and Muyshondt 1978; Dutra et al. 2006). Females lay pale green and white banded eggs that hatch five days after being laid (Muyshondt and Muyshondt 1978; Dutra et al. 2006). Females choose to lay eggs on nettle plants lacking fruit to reduce ant attraction, thereby reducing *S. blomfildia* larval mortality from ant attacks (Machado and Freitas 2001). Originally described as a member of the genus *Papilio* (Fabricius 1781), *S. blomfildia* has been referred to by other specific epithets including *S. bella* and *S. pluto*, now considered junior synonyms (Muyshondt and Muyshondt 1978). Here we report the complete mitochondrial genome (mitogenome) sequence of *S. blomfildia* from specimen Sb2017.1, collected in Tingo Maria, Peru (GPS 9.29616S, 75.99831 W) in October 2017 that has been pinned, spread, and deposited in the Wallis Roughley Museum of Entomology, University of Manitoba (http://www.wallisroughley.ca/, Jason Gibbs, Jason.Gibbs@umanitoba.ca) voucher WRME0507738.

DNA was prepared from a specimen leg using a DNeasy Blood and Tissue kit **(**Qiagen, Düsseldorf, Germany) with slight modifications to the standard protocol as described in McCullagh and Marcus (2015). DNA was sheared by sonication and a fragment library was prepared using the NEBNext Ultra II DNA Library Prep Kit for Illumina (New England Biolabs, Ipswich, Massachusetts) as previously described (Peters and Marcus 2017), before sequencing by Illumina NovaSeq6000 (San Diego, California) (Marcus 2018). The mitogenome of *S. blomfildia* (Genbank MZ151338) was assembled and annotated using Geneious Prime 2021.1.1 from an SRA library of 18,400,288 paired 150 bp reads (Genbank SRA PRJNA729786) using *a Baeotus beotus* reference mitogenome (Lepidoptera: Nymphalidae, MW566598) (Lalonde 2021). The *S. blomfildia* nuclear rRNA repeat (Genbank MZ198233) was also assembled and annotated using a *B. beotus* (MW571038) reference sequence. The rRNA repeat sequence is increasingly recognized as being very useful for phylogenetic comparisons based on nuclear markers (Dodsworth 2015; Coissac et al. 2016; Marcus 2018; Krehenwinkel et al. 2019), so we have chosen to release it here.

The *S. blomfildia* circular 15,149 bp mitogenome assembly was composed of 24,120 paired reads with nucleotide composition: 34.5% A, 10.6% C, 5.5% G, and 49.4% T. The gene composition and order in *S. blomfildia* is typical of the arrangement found in most butterfly mitogenomes (Park et al. 2016). The *S. blomfildia* protein coding gene start codons include: ATG (*ATP6*, *COX2*, *COX3*, *CYTB*, *ND1*, *ND4*, *ND4L*), ATT (*ATP8, ND2, ND5, ND6*), ATC, (*ND3*), and CGA, an atypical *COX1* start codon that is also found in the *COX1* gene of many other insects (Liao et al. 2010). The mitogenome contains four protein-coding genes (*COX1, COX2, ND4 ND5*) with single-nucleotide (T) stop codons, and four protein-coding gene (*ATP6, CYTB, ND1 ND3*) with two-nucleotide (TA) stop codons completed by post-transcriptional addition of 3′ A residues. All structures of the tRNAs were verified using ARWEN v.1.2 (Laslett and Canback 2008) and have typical cloverleaf secondary structures with the exception for trnS (AGN) where the dihydrouridine arm is replaced by a loop, whereas the control region and mitochondrial rRNAs are typical for Lepidoptera (McCullagh and Marcus 2015).

Phylogenetic reconstruction (Figure 1) was completed using the complete mitogenome of *S. blomfildia* and 37 other mitogenomes from the family Nymphalidae. Sequences were aligned in CLUSTALX 2.1 (Thompson et al. 1997; Larkin et al. 2007) and analyzed using Bayesian Inference with the GTR + I + G model (model selected using jModeltest 2.1.1 (Darriba et al. 2012)) in Mr. Bayes version 3.2.7 (Ronquist and Huelsenbeck 2003; Ronquist et al. 2012). Phylogenetic analysis places *Smyrna* as a member of the tribe Nymphalini and sister to a clade containing genera *Araschnia*, *Vanessa*, *Polygonia*, and *Aglais*, confirming the findings of some previous molecular phylogenetic analyses (Wahlberg et al. 2005; Wahlberg and Wheat 2008). Placing *Smyrna* in tribe Nymphalini is also supported by larval morphological characters (Muyshondt and Muyshondt 1978). This differs from the classical taxonomic placement of *Smyrna* with *Baeotus beotus* in the tribe Coeini based on adult morphology (Muyshondt and Muyshondt 1978) and supported by a different molecular phylogenetic analysis (Wahlberg et al. 2009). Based on our results, we agree with prior researchers who have reclassified *Smyrna* in tribe Nymphalini.

**Figure 1.**
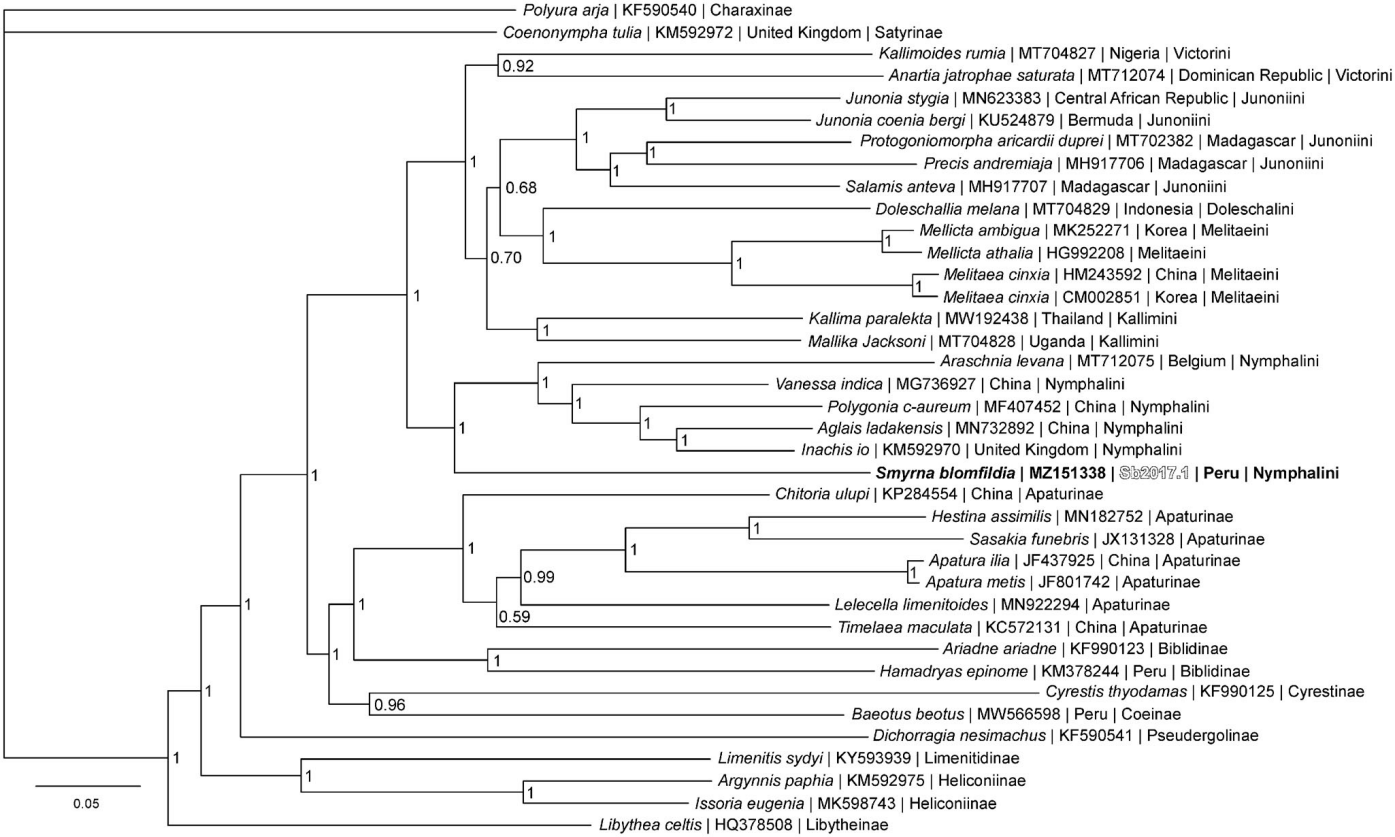
The Bayesian phylogeny (GTR + I + G model, average Potential Scale Reduction Factor (PSRF) = 1, average deviation of split frequencies = 0.001523) of the *Smyrna blomfildia* mitogenome, 37 additional mitogenomes from within family Nymphalidae, including outgroup species *Polyura arja* (Charaxinae) and *Coenonympha tullia* (Satyrinae) (Alexiuk et al. 2020; Hamilton et al. 2020; Lalonde and Marcus 2020; Payment et al. 2020; Lalonde 2021), produced by 10 million MCMC generations in MrBayes, with sampling every 100 generations, and after discarding the first 250,000 generations as burn-in. At each node, the Bayesian posterior probability values determined by MrBayes are given.

## Data Availability

The genome sequence data that support the findings of this study are openly available in GenBank of NCBI at [https://www.ncbi.nlm.nih.gov] (https://www.ncbi.nlm.nih.gov/) under the accession nos. MZ151338 and MZ198233. The associated BioProject, SRA, and Bio-Sample numbers are PRJNA729786, SRX10874928, and SAMN19163223 respectively.
